# Inhaled Corticosteroid Use and Self-Reported Voice Problems in a Nordic Population-Based Cohort

**DOI:** 10.2147/JAA.S638697

**Published:** 2026-10-03

**Authors:** Sofia Ögefeldt, Greta Öhlund Wistbacka, Ellika Schalling, Henrik Johansson, Andrei Malinovschi, Christer Janson, Helena Backman, Mathias Holm, Ane Johannessen, Caroline Ahlroth Pind, Elin Dahlén, Össur Ingi Emilsson

**Affiliations:** 1 Department of Public Health and Caring Sciences; Speech-Language Pathology, Uppsala University, Uppsala, Sweden; 2 Department of Medical Sciences, Respiratory, Allergy and Sleep Research, Uppsala University, Uppsala, Sweden; 3 Department of Geriatrics, Rehabilitation, Pain Centre and Palliative Care, Uppsala University Hospital, Uppsala, Sweden; 4 Department of Women’s and Children’s Health: Physiotherapy and Behavioral medicine, Uppsala University, Uppsala, Sweden; 5 Department of Medical Sciences, Clinical Physiology, Uppsala University, Uppsala, Sweden; 6 Department of Public Health and Clinical Medicine, the OLIN and Sunderby Research Unit, Umeå University, Umeå, Sweden; 7 Occupational and Environmental Medicine, School of Public Health and Community Medicine, Institute of Medicine, Sahlgrenska Academy, University of Gothenburg, Gothenburg, Sweden; 8 Department of Global Public Health and Primary Care, University of Bergen, Bergen, Norway; 9 Centre for Clinical Research Västmanland, Uppsala University, Västerås, Sweden; 10 Department of Pharmacy; Drug Epidemiology, Uppsala University, Uppsala, Sweden; 11 The NEPI Foundation-Network for Pharmacoepidemiology, Stockholm, Sweden; 12 Faculty of Medicine, University of Iceland, Reykjavik, Iceland

**Keywords:** asthma, inhaled corticosteroids, chronic cough, voice problems, dysphonia, hoarseness, ICS, asthma symptoms

## Abstract

**Background:**

Voice problems are frequently reported among users of inhaled corticosteroids (ICS), but it remains unclear whether these problems reflect pharmacological effects, asthma, or comorbid chronic cough.

**Aim:**

This study aimed to examine the association between ICS use and voice problems in a Nordic population-based cohort, accounting for asthma symptoms and chronic cough. Secondary aims included dose–response patterns and differences by inhaler device type.

**Methods:**

Cross-sectional data from adults in Sweden and Norway participating in the Respiratory Health in Northern Europe IV study (RHINE, n=5,986) were linked to national prescription registers. ICS exposure during 2021 was quantified using annual filled defined daily doses (FDDD), calculated from dispensed prescriptions based on the corticosteroid content per inhalation and number of dispensed doses. Associations between self-reported voice problems and ICS use, asthma symptoms, and chronic cough were analyzed using logistic regression, adjusted for multiple confounders. Dose–response, exposure overlap, and inhaler device type were examined in secondary analyses.

**Results:**

Voice problems increased with higher ICS exposure, from 15.5% among non-users to 59.7% in the highest dose category. The dose–response pattern showed a steep increase at lower doses with attenuation at higher exposure levels. In adjusted analyses including ICS use, asthma symptoms and chronic cough, ICS use remained associated with voice problems (aOR 1.54 [1.13–2.10] for 1–150 FDDD; 2.88 [1.98–4.21] for 151–300 FDDD; and 3.00 [2.03–4.43] for >300 FDDD); as did asthma symptoms (aOR 2.31 [1.90–2.82] for one symptom; 3.14 [2.45–4.03] for ≥2 symptoms); and chronic cough (aOR 2.03 [1.67–2.46]). The highest prevalence was observed among participants with overlapping ICS use, asthma symptoms, and chronic cough. Inhaler device type did not affect voice problems, with reservation for low statistical power.

**Conclusion:**

ICS use, asthma symptoms, and chronic cough were independently associated with voice problems, with a clear dose–response for ICS, underscoring the need to consider both medication and respiratory symptoms in clinical assessment.

## Background

Asthma is a common chronic respiratory disease characterized by airway inflammation and variable airflow obstruction, resulting in fluctuating symptoms such as wheeze, breathlessness, chest tightness, and cough.^1^,^2^ Higher symptom burden typically reflects poorer asthma control or more severe disease and is strongly associated with impaired health-related quality of life.^1^,^3^

Inhaled corticosteroids (ICS) are the cornerstone of asthma treatment, and are recommended for all patients, including those with mild disease.^4^ They are also used in selected cases of chronic obstructive pulmonary disease (COPD).^5^ Although ICS are generally safe and effective,^6^ local laryngeal side effects such as dysphonia, throat discomfort and dryness are frequently reported.^6–11^ These adverse effects may contribute to reduced treatment adherence and increased disease burden.^12–14^

ICS-related laryngeal side effects are thought to result from corticosteroid deposition on the vocal fold mucosa, potentially causing irritation, inflammation, or candidiasis.^7^ Factors such as dose, drug formulation, particle size, inhaler type, and inhalation technique, may influence the extent of oropharyngeal deposition.^7^,^9^,^10^

Although ICS are often described as the primary cause of voice problems in individuals with asthma, the disease itself may also contribute.^15^,^16^ Bronchoconstriction and increased respiratory effort can reduce breath support for phonation,^7^,^17^ while airway inflammation may promote coughing and throat clearing that mechanically irritate the vocal folds.^7^,^8^ Chronic cough, commonly associated with asthma,^18^ has also been linked to voice problems.^19^ Together, these mechanisms imply that asthma-related factors may contribute to vocal symptoms independently of ICS exposure.

Because higher ICS doses are typically prescribed to individuals with more symptomatic or difficult-to-control asthma,^4^ previously reported dose-response associations between ICS and voice problems^9^ are challenging to interpret. Voice problems in conjunction with higher ICS doses may reflect medication effects, more severe underlying disease, or related conditions such as chronic cough. This interaction complicates the distinction between treatment-related effects and underlying disease factors. Therefore, population-based studies that consider asthma control and cough are needed to clarify the relationship between ICS use and voice problems.

This study aimed to investigate the association between ICS use and self-reported voice problems in a Nordic population-based cohort. A key objective was also to assess whether any found association between ICS use and voice problems can be explained by asthma symptoms and related conditions, such as chronic cough, or whether it reflects effects of ICS treatment itself. By addressing these aspects, the study aims to clarify the relative contribution of asthma, chronic cough, and ICS treatment to voice problems.

## Method

This cross-sectional, register-based study used data from the Swedish and Norwegian study centers participating in the fourth wave of the Respiratory Health in Northern Europe study (RHINE IV). RHINE is a multicenter longitudinal cohort study initiated in 1990, involving a randomly selected population sample (n = 21,673) born between 1945 and 1973.^20^,^21^ Participants have completed questionnaires about exposures and respiratory health approximately every 10 years. The study is conducted across seven participating northern European centers: Aarhus (Denmark); Umea, Gothenburg, and Uppsala (Sweden); Bergen (Norway); Reykjavik (Iceland); and Tartu (Estonia). Data collection for RHINE IV took place between 2021 and 2022. In total, 6,165 individuals, aged 49–76 years, completed the Swedish and Norwegian questionnaire; 5,986 had complete data on ICS use and self-reported voice problems and were therefore included in the analyses. The restriction to Swedish and Norwegian participants was due to availability of prescription register data on ICS in these countries.

The RHINE IV questionnaire includes items concerning various aspects of respiratory health, such as asthma and cough, as well as patient-related factors including sex, age, height, weight, educational level, and smoking history. In this wave, a question concerning subjective voice problems was also incorporated, previously utilized and validated in a Swedish prevalence study.^22^ The complete RHINE IV questionnaire is available online.^23^

All participants provided written informed consent, and the study was approved by the Swedish Ethical Review Authority (Dnr 2020–05053 and 2022–05385-02) and the Regional Committee for Medical Research Ethics of Western Norway (REK West) (Dnr 236168 and 300765).

### ICS Use

To examine associations between ICS use and self-reported voice problem, individual-level RHINE IV questionnaire data were linked to the Swedish and Norwegian National Prescribed Drug Registers, which captures all pharmacy-dispensed prescriptions nationwide. ICS use during 2021 was estimated from filled prescriptions and expressed as filled defined daily doses (FDDD). The defined daily dose (DDD) is established by the World Health Organization, and represents the assumed average maintenance dose per day for a drug used for its main indication in adults.^24^

As the prescription registers usually record the FDDD content of combination inhalers (for example fixed-dose ICS-LABA) based on the bronchodilator dose rather than the corticosteroid dose, all prescriptions were evaluated for their FDDD content of ICS specifically. To calculate ICS-specific FDDD, the ICS content per dose (measured in µg) was multiplied by the number of dispensed doses in each prescription and then divided by the WHO-defined DDD (in µg) for the corresponding ICS.^24^ For fluticasone furoate, for which no WHO-defined DDD was available, a study specific DDD was based on GINA guidelines.^4^ This approach allowed combination inhalers to be classified according to ICS exposure rather than the bronchodilator component. The ICS substances and DDD values used in these calculations are shown in Supplementary Table S1.

For analysis, ICS use was operationalized as a binary variable (any vs no ICS use) for descriptive statistics, as a categorical variable reflecting increasing annual exposure (0, 1–150, 151–300, and >300 FDDD/year), and as a continuous variable. Analyses were restricted to participants with complete RHINE IV data and successful register linkage.

#### ICS Device Type

Inhaler device type was determined based on ICS device type prescribed during 2021, classified as either pressurized metered-dose inhalers (pMDI) or dry powder inhalers (DPI). Participants were classified as pMDI users if they had been prescribed ICS use via pMDI only, and as DPI users if they had been prescribed DPI use only. Those prescribed both device types were classified as mixed users, and those prescribed no ICS were classified as non-users. The resulting variable therefore comprised four categories: 0 = none, 1 = pMDI, 2 = DPI, and 3 = both.

### Voice Problems

Participants were considered to have a subjective voice problem if they responded with either a 2 or a 3 to the question: Does your voice tire, strain, or get hoarse when you talk? Disregard symptoms that depend on current cold or upper-airway infection. The voice symptoms may vary but try to estimate an average. The response option was: *1 = No; 2 = Yes, to a small extent; 3 = Yes, to a great extent*. For most analyses, individuals who reported voice problems to any extent (responses 2 or 3) were grouped together due to small group sizes across ICS dose categories.

### Asthma Symptoms and Cough

To account for current asthma-related symptoms, reflecting possible insufficient disease control or undiagnosed asthma, the following symptoms during the past 12 months were derived from four RHINE IV items: (I) attack of asthma, (II) wheeze/whistling in the chest, (III) waking with chest tightness, and (IV) waking with shortness of breath. These items have been used in prior studies to capture asthma-related symptoms in the general population, including individuals without a physician-diagnosed asthma.^20^,^25–27^ Asthma symptoms were operationalized in two ways: a binary indicator (any vs none) for descriptive statistics, and an ordinal three-level variable for regression models coded 0 = no symptoms, 1 = one symptom, 2 = two to four symptoms during the past 12 months. Asthma-related symptoms were derived for the full cohort regardless of asthma diagnosis and were also summarized separately for participants with self-reported physician-diagnosed asthma (“Have you ever had asthma diagnosed by a doctor?”).

For exploratory secondary analyses, a four-category variable was created by combining physician-diagnosed asthma with current asthma symptoms to distinguish between (0) no physician-diagnosed asthma and no symptoms, (1) no physician-diagnosed asthma but current symptoms, (2) physician-diagnosed asthma without current symptoms, and (3) physician-diagnosed asthma with current symptoms. This variable allowed exploration of the relative influence of reported asthma diagnosis and symptom burden in the association between ICS use and voice problems.

Chronic cough was defined as an affirmative response to the question “In recent years, have you been troubled by a protracted cough?”. This definition has been used in previous studies.^19^,^28^

### Confounders

Confounders were selected by constructing a directed acyclic graph (DAG) incorporating variables recognized in prior research as potential confounding factors associated both with ICS use and voice problems (se Supplementary Figure S1). The confounders included study center, sex, age, body mass index (BMI), smoking history, chronic rhinosinusitis (CRS), COPD, nocturnal gastroesophageal reflux (nGER), current anxiety and/or depression, hay fever/nasal allergies, and exposure to mold or dampness in the home environment. Study center referred to the participation centers (Sweden: Uppsala, Gothenburg, and Umea; Norway: Bergen). Sex was self-reported as male or female. Age and BMI were calculated from self-reported data on birth year and height and weight (kg/m^2^). COPD was based on a self-reported physicians’ diagnosis. nGER was defined as heartburn or belching occurring during sleep at least once per week.^29^ CRS was defined according to the European Position Paper on Rhinosinusitis and Nasal Polyps (EPOS) criteria,^30^ based on the presence of at least two of the following symptoms lasting for more than 12 consecutive weeks during the past year: nasal blockage, nasal discharge, facial pain or pressure, and reduced sense of smell, where at least one of the symptoms had to be nasal blockage or nasal discharge. Current anxiety and/or depression were based on self-reported current treatment for these conditions. Hay fever/nasal allergies were defined as a positive response to the question “Do you have any nasal allergies including hay fever?”. Exposure to mold or dampness was defined as any positive response to items assessing water leakage or water damage, floor discoloration or bubbling, or visible indoor mold during the past 12 months.^31^ Educational level (categorized as elementary school, high school, or university) was evaluated as a potential confounder but was not included in the final multivariable models because of substantial missing data; it was, however, included in a later complete case sensitivity analysis.

### Statistical Analysis

Descriptive characteristics of the study population by ICS dose category were summarized using means, standard deviations, and proportions. Overlap between ICS use, asthma symptoms and chronic cough was visualized using Venn diagrams, and the prevalence of voice problems within each subgroup was assessed descriptively.

Logistic regression models were used to estimate odds ratios (ORs) with 95% confidence intervals (CIs) for the association of self-reported voice problems with ICS use, asthma symptoms and chronic cough, with results presented both before and after adjustment for confounders (age, sex, BMI, smoking history, study center, anxiety/depression, nGER, COPD, CRS, hay fever/nasal allergies, and exposure to mold or dampness in the home environment), to evaluate the individual contributions of ICS, asthma symptoms and chronic cough to voice problems.

To evaluate whether inhaler device type contributed to voice problems beyond ICS dose; device type was included in models adjusted for ICS dose category and all covariates.

The association between voice problems and ICS dose as a continuous variable was modelled using logistic regression with restricted cubic splines, with 0 FDDD (no ICS use) as the reference category. Knots were placed at 50, 150 and 600 FDDD, corresponding approximately to the 10th, 50th and 95th percentiles of the ICS dose distribution among ICS users within the observed dose range ≤900 FDDD. Because very few participants had doses above 900 FDDD, which resulted in unstable spline estimates, the dose–response function was visualized only within this range. Adjusted dose–response odds ratios with 95% confidence intervals were derived from the spline model.

Effect modification by asthma symptom burden was assessed by including an interaction term between ICS use and asthma symptoms. In addition, a secondary stratified analysis was also conducted using the four-category asthma variable defined earlier, which combines self-reported doctor-diagnosed asthma and current asthma symptoms.

Statistical significance was defined as *p* < 0.05, but results were primarily interpreted based on the magnitude and direction of associations. Analyses were conducted using Stata version 19.5 (StataCorp, TX, USA).

## Results

### Participant Characteristics

Most participants were non-users of ICS (n = 5,401; 90%), and among those with ICS, the largest proportion was observed in the lowest dose category, with lower proportions in the higher dose categories (Table 1). Baseline characteristics differed across these groups. In the higher-dose groups, participants more often reported two or more asthma symptoms and a higher prevalence of doctor-diagnosed asthma.

**Table 1:** Baseline Characteristics of the Study Population Stratified by Inhaled Corticosteroid (ICS) Dose Category (FDDD 2021)

	Total	No ICS	1-150 FDDD	151-300 FDDD	>300 FDDD	Missing
**N (%)**	5,986 (100%)	5,401 (90.2%)	268 (4.5%)	163 (2.7%)	154 (2.6%)	
**Age (years, mean (SD))**	63 (7.2)	63 (7.2)	64 (7.1)	64 (7.1)	64 (7.1)	-
**BMI (kg/m^2^, mean (SD))**	26.4 (4.4)	26.3 (4.2)	27.5 (5.2)	27.3 (5.4)	27.8 (5.2)	81 (1.35%)
**Sex**						-
Male	2,871 (48.0%)	2,634 (48.8%)	110 (41.0%)	68 (41.7%)	59 (38.3%)	
Female	3,115 (52.0%)	2,767 (51.2%)	158 (59.0%)	95 (58.3%)	95 (61.7%)	
**Smoking status**						66 (1.10%)
Never	3,235 (54.6%)	2,947 (55.1%)	147 (56.1%)	76 (47.2%)	65 (42.5%)	
Former	2,276 (38.4%)	2,030 (38.0%)	102 (38.9%)	72 (44.7%)	72 (47.1%)	
Current	409 (6.9%)	367 (6.9%)	13 (5.0%)	13 (8.1%)	16 (10.5%)	
**Educational level**						1065 (17.8%)
Elementary school	435 (8.8%)	373 (8.4%)	27 (12.5%)	19 (14.0%)	16 (11.9%)	
High school	2,003 (40.7%)	1,816 (40.9%)	83 (38.4%)	52 (38.2%)	52 (38.8%)	
College/university	2,483 (50.5%)	2,246 (50.6%)	106 (49.1%)	65 (47.8%)	66 (49.3%)	
**Study center**						-
Bergen	1,513 (25.3%)	1,392 (25.8%)	27 (10.1%)	24 (14.7%)	70 (45.5%)	
Gothenburg	1,286 (21.5%)	1,164 (21.6%)	65 (24.3%)	34 (20.9%)	23 (14.9%)	
Umea	1,501 (25.1%)	1,336 (24.7%)	84 (31.3%)	49 (30.1%)	32 (20.8%)	
Uppsala	1,686 (28.2%)	1,509 (27.9%)	92 (34.3%)	56 (34.4%)	29 (18.8%)	
**Chronic cough**	842 (14.2%)	610 (11.4%)	105 (39.6%)	67 (41.1%)	60 (39.0%)	49 (0.82%)
**Asthma symptoms**						2 (0.03%)
None	4,530 (75.7%)	4,390 (81.3%)	69(25.7%)	38 (23.3%)	33 (21.4%)	
1 symptom	916 (15.3%)	702 (13.0%)	110 (41.0%)	59 (36.2%)	45 (29.2%)	
2 or more symptoms	538 (9.0%)	307 (5.7%)	89 (33.2%)	66 (40.5%)	76 (49.4%)	
**Doctor diagnosed asthma**	778 (13.2%)	362 (6.8%)	156 (60.9%)	126 (81.3%)	134 (88.7%)	106 (1.77%)
**Anxiety/depression**	1,104 (19.0%)	952 (18.2%)	67 (25.8%)	39 (24.7%)	46 (30.3%)	178 (2.97%)
**Mold or dampness at home**	400 (6.7%)	360 (6.7%)	25 (9.4%)	12 (7.4%)	3 (1.9%)	47 (0.79%)
**Hay fever/nasal allergies**	1,617 (27.3%)	1,347 (25.2%)	121 (45.5%)	77 (48.1%)	72 (46.8%)	56 (0.94%)
**CRS**	320 (5.5%)	223 (4.2%)	45 (17.5%)	24 (15.0%)	28 (18.9%)	146 (2.44%)
**COPD**	165 (2.8%)	88 (1.7%)	23 (8.7%)	24 (15.0%)	30 (19.9%)	79 (1.32%)
**nGER**	562 (9.8%)	477 (9.2%)	38 (15.0%)	28 (18.4%)	19 (12.9%)	253 (4.23%)

**Notes**: Population: Participants with available data on ICS dispensing (FDDD 2021) and voice problems (n 5,986). Data are presented as % or mean ± Standard Deviation (SD).

**Abbreviations**: BMI, Body Mass Index; CRS, Chronic Rhinosinusitis; COPD, Chronic Obstructive Pulmonary Disease; nGER, Nocturnal Gastroesophageal Reflux.

Chronic cough, CRS, reflux and hay fever were all more common among ICS users than non-users; however, their prevalence varied across the ICS dose categories without a consistent dose-response pattern. In contrast, COPD was distinctly more frequent among those receiving larger amounts of ICS, with prevalence increasing from 1.7% in non-users to 19.9% in the highest dose group.

Study center distributions differed across ICS groups, with a higher proportion of participants from Bergen in the highest dose category. Overall, missingness was low for most variables in Table 1. The only variable with a notable degree of missing data was educational level, which therefore was excluded from the main multivariable models.

### Proportion of Voice Problems Across Overlapping ICS, Asthma Symptoms and Cough Groups

A clear overlap was observed between ICS use, asthma symptoms, and chronic cough (Figure 1). The analysis included 5,936 participants, of whom 4,030 had none of these factors (subgroup sample sizes are provided in Supplementary Figure S2). The proportion of participants reporting voice problems differed substantially between these subgroups. Voice problems were most frequent in participants with overlapping ICS use and asthma symptoms (48.3%), and highest among those with all three characteristics (65.3%). The prevalence of voice problems was lower in participants with only chronic cough (23.9%) or only asthma symptoms (27.6%) and lowest in those outside all three categories (10.8%) (Figure 1).

**Figure 1:**
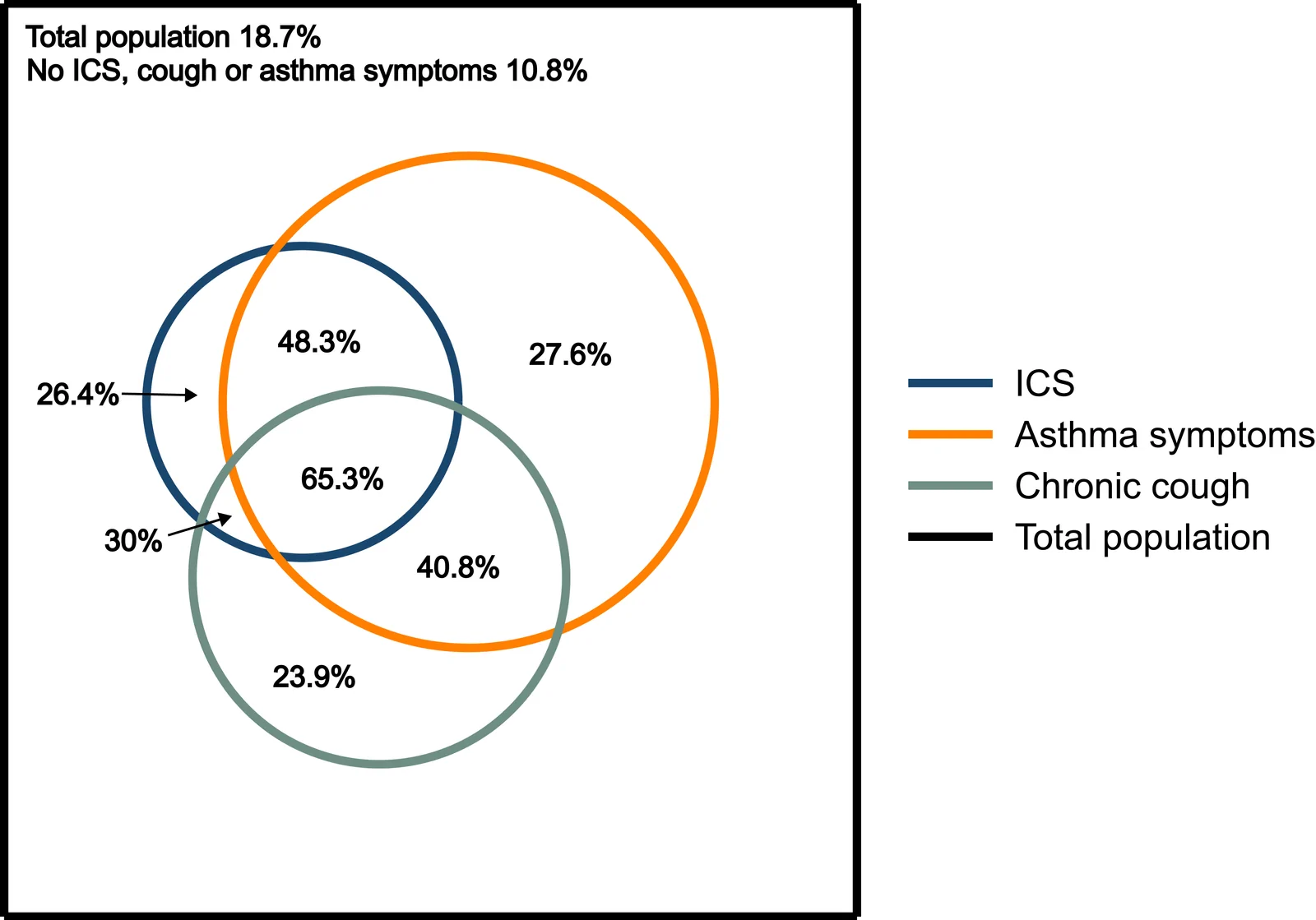
Prevalence of self-reported voice problems across ICS, asthma symptoms, and chronic cough subgroups, expressed as the proportion (%) of participants reporting voice problems within each subgroup and their overlaps. The overall prevalence in the total study population, as well as among participants with none of these factors, is shown outside the circles.

When restricting the asthma symptom group to participants with physician-diagnosed asthma, similar patterns were observed. This analysis included 5,832 participants, of whom 4,550 had none of these factors. Voice problems remained more common among those with both asthma symptoms and ICS use (50.3%) and highest in the triple-overlap group (70.0%), whereas prevalence was lower in groups defined by chronic cough or ICS use alone (Subgroup prevalences and sample sizes are presented in Supplementary Figure S3).

### Association Between ICS and Voice Problems

The prevalence of voice problems increased with higher annual ICS doses (Table 2), from 38.1% in the lowest dose category (1–150 FDDD) to 56.4% in the mid-dose group (151–300 FDDD) and 59.7% among those with >300 FDDD. A broadly similar pattern was observed when voice problems were analyzed by degree of severity (Supplementary Table S2). Higher prevalence of voice problems was also observed with increasing numbers of asthma symptoms and among individuals with chronic cough (Table 2).

**Table 2:** Prevalence and Unadjusted Odds Ratios for Voice Problems by ICS Dose, Asthma Symptoms and Cough

Exposure	Total n	Voice Problems, n	Voice Problems, %	Unadjusted OR	95% CI	p-value
**ICS dose categories**						
No ICS	5,401	835	15.5%	ref		
1–150 FDDD	268	102	38.1%	3.36	2.60–4.35	< 0.0001
151–300 FDDD	163	92	56.4%	7.08	5.15–9.74	< 0.0001
>300 FDDD	154	92	59.7%	8.11	5.83–11.29	< 0.0001
**Number of asthma symptoms**						
No symptom	4,530	555	12.3%	ref		
1	916	294	32.1%	3.39	2.87–3.99	< 0.0001
2 or more	538	272	50.6%	7.32	6.05–8.86	< 0.0001
**Chronic cough**						
No cough	5,095	777	15.3%	ref		
Cough	842	336	39.9%	3.70	3.15–4.32	< 0.0001

**Notes**: Values are presented as total n, number with voice problems, and prevalence (%). Odds ratios (ORs) with 95% confidence intervals (CIs) are unadjusted and estimated using logistic regression. The reference categories were no ICS use, no asthma symptoms, and no chronic cough, respectively. ICS dose categories are based on annual filled defined daily doses (FDDD).

In multivariable models adjusted for asthma symptoms, chronic cough, and confounders, ICS use remained positively associated with self-reported voice problems. Higher ICS doses were associated with progressively higher odds of reporting voice problems (Table 3). Asthma symptoms and chronic cough were also independently associated with voice problems, but their inclusion in the model did not attenuate the positive association between ICS exposure and voice problems. Increasing numbers of asthma symptoms were also associated with higher odds of voice problems.

**Table 3:** Adjusted Logistic Regression Models for the Association Between ICS Use and Voice Problems

Exposure	OR	95% CI	p-value
**ICS dose categories (n 5341)**			
1-150 FDDD	1.54	1.13–2.10	0.006
151-300 FDDD	2.88	1.98–4.21	<0.0001
>300 FDDD	3.00	2.03–4.43	<0.0001
**Number of asthma symptoms**			
1	2.31	1.90–2.82	<0.0001
2 or more	3.14	2.45–4.03	<0.0001
**Chronic cough**	2.03	1.67–2.46	<0.0001

**Notes**: Logistic regression adjusted for asthma symptoms, chronic cough, age, sex, BMI, smoking history, CRS, COPD, nGER, hay fever/nasal allergies, anxiety/depression, mold or dampness at home, and study center. Results are presented as OR; 95% CI.

Including educational level in the models did not meaningfully change the estimates but substantially reduced the number of participants due to missing data; models are therefore presented without this variable.

When ICS dose was modelled as a continuous exposure using restricted cubic splines and adjusted for potential confounders, the dose–response curve showed a gradual increase in the odds of voice problems compared with 0 FDDD, with the steepest rise occurring between low and moderate doses (Figure 2). The curve continued to increase up to approximately 400–500 FDDD, after which it appeared to plateau. At the upper end of the supported dose range (≤900 FDDD), the confidence intervals widened due to fewer observations. Overall, the spline-based dose–response analysis indicated that higher ICS doses were associated with increased odds of voice problems, particularly at low and intermediate dose levels, with a tendency towards a plateau at higher doses.

**Figure 2:**
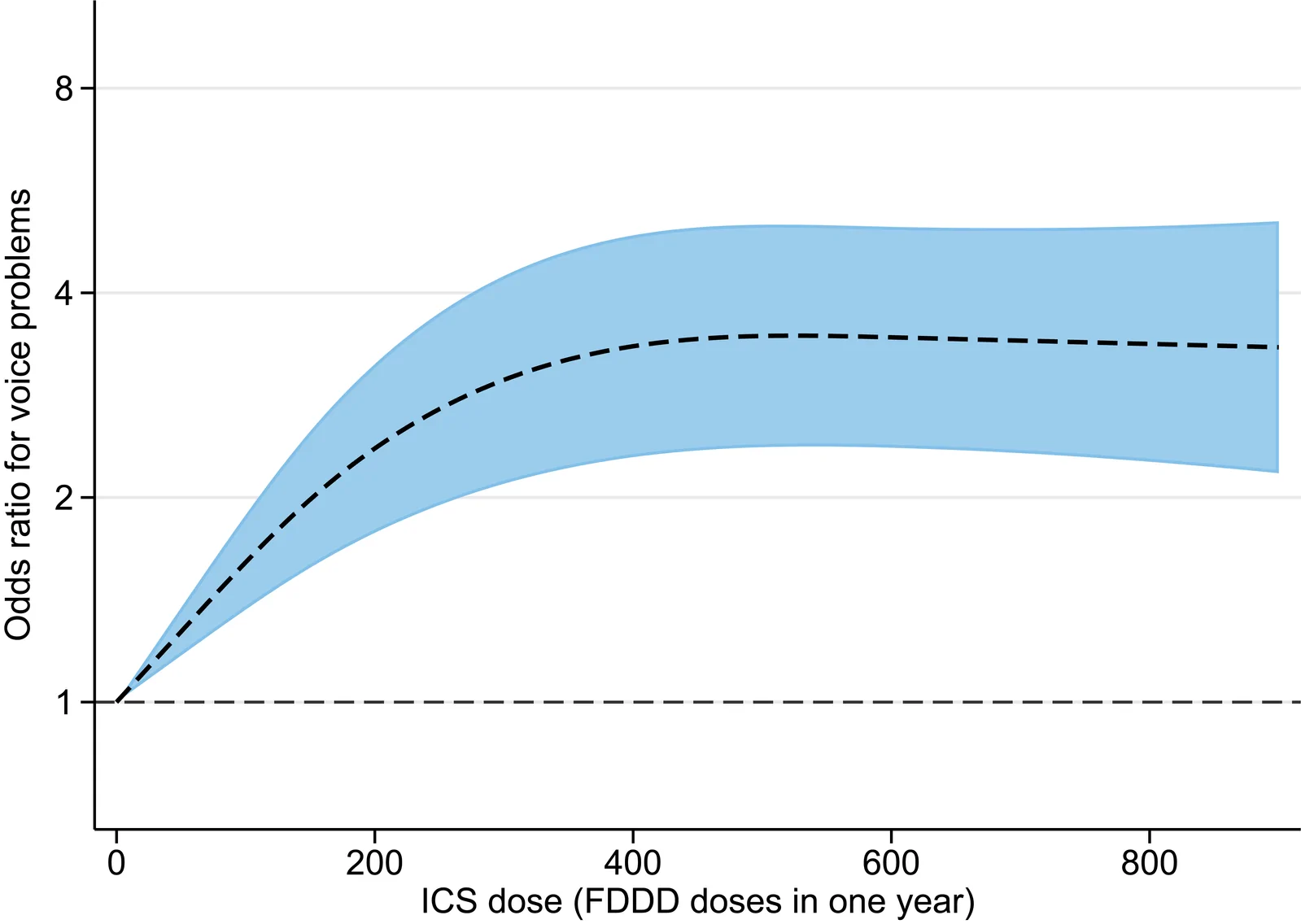
Adjusted odds ratio for voice problems across ICS dose range. Estimated using logistic regression with restricted cubic splines (knots at 50, 150 and 600 FDDD; reference = 0 FDDD). The curve is shown up to 900 FDDD, reflecting the dose range with adequate data support. The dotted line indicates the estimated dose–response function and the shaded area, the 95% confidence interval. The model was adjusted for asthma symptoms, chronic cough, age, sex, BMI, smoking history, CRS, COPD, nGER, hay fever/nasal allergies, anxiety/depression, mold or dampness at home, and study center.

Analyses restricted to participants with physician-diagnosed asthma and asthma symptoms yielded estimates for ICS dose categories, asthma symptoms, and chronic cough that were comparable in magnitude to those observed in the main analysis (Supplementary Table S3).

No evidence of interaction between ICS dose categories and asthma symptom levels was observed in either the full population (*p* = 0.77) or in analyses restricted to participants with doctor-diagnosed asthma (*p* = 0.17).

A four-category variable combining physician-diagnosed asthma with current symptoms was used in an exploratory subgroup analysis. These stratified estimates were broadly consistent with the main models, and inclusion of the four-category asthma variable did not materially change the association between ICS use and voice problems (Supplementary Table S4).

### Mode of Administration (Spray vs Dry Powder)

ICS device types were unevenly distributed, with most users receiving DPIs (n = 390), fewer using pMDIs (n = 55), and only a small group using both device types (n = 19). Use of DPIs was associated with voice problems in models not adjusted for ICS dose. However, no association between device type and voice problems remained after adjustment for ICS dose (Supplementary Table S5).

## Discussion

In this population-based sample of adults aged 49–76 in Sweden and Norway, ICS use was strongly associated with self-reported voice problems. Voice problems increased with increasing ICS doses, most pronounced at low doses, and plateaued at higher doses. In participants without ICS use, approximately one in six reported voice problems compared with nearly three in five in the highest dose category. Asthma symptoms and chronic cough were also independently associated with voice problems, and the association between ICS exposure and voice problems was similar among participants with both physician-diagnosed asthma and symptoms and those with only symptoms. Finally, substantial overlap was observed between ICS use, asthma symptoms, and chronic cough. Among participants, where these three factors co-occurred, reported voice problems exceeded 60%.

In the present study, about half of all ICS users reported voice problems, with an even greater prevalence in higher dose categories. This prevalence is in the higher range of those previously reported,^7^,^8^,^11^,^32–35^ which may partly relate to differences in outcome measurements. Voice problems were assessed using a validated symptom question encompassing hoarseness, vocal fatigue, and strain, which may have increased the sensitivity for detecting patient-experienced voice difficulties.^22^,^36^ In fully adjusted models ICS still showed a dose-dependent association with voice problems. A recent systematic review highlighted that many previous observational studies of ICS-related voice outcomes have adjusted for a limited set of potential confounders or not reported such adjustments at all.^11^ By adjusting for relevant respiratory conditions and comorbidities, the present study allows a clearer distinction between ICS-related effects and underlying disease burden, while providing population-level estimates consistent with previous evidence of ICS-related voice problems.

In addition to the overall association with ICS, a clear dose–related pattern was found, with increasing odds of voice problems at low to moderate doses followed by a plateau at higher exposure levels. This pattern is broadly consistent with previous studies based on prescribed daily dose,^9^,^37^,^38^ while extending the evidence to cumulative exposure estimated from dispensed prescriptions in a population-based sample.

Asthma symptoms were also independently associated with voice problems in the present study. A limited number of studies have examined this connection. In a population-based study of nearly 19,000 adults, Park and Choi^15^ reported increased odds of voice problems both among individuals with asthma who had not used medication during the previous year (aOR 1.76) and among those who had (aOR 2.60). However, medication type was not specified, and it remains unclear whether non-medicated participants had active symptoms, prior asthma, or untreated current disease, limiting the ability to distinguish treatment-related effects from disease related factors. A smaller clinical study by Asnaashari et al^16^ found perceptual and acoustic voice differences in untreated asthma patients compared to healthy controls, suggesting that asthma-related symptom activity itself may influence voice characteristics.

Several mechanisms may help explain the independent associations of ICS use, asthma symptoms, and cough with self-reported voice problems observed in this study. Regarding ICS exposure, local effects on the laryngeal mucosa have been proposed, such as irritation, mucosal dryness, and vascular changes.^10^,^34^,^39^ Less common ICS related effects, such as vocal fold myopathy and laryngeal candidiasis, have also been reported.^34^,^39–41^ Inhaler-related factors, including drug formulation, particle size, and device characteristics may also influence exposure and thereby contribute to voice problems.^7^ However, in the present cohort, inhaler type did not materially affect the overall association once ICS dose was taken into account. Estimates for some device subgroups were imprecise because of small subgroup sizes and should therefore be interpreted with caution.

In asthma, in addition to mechanisms such as airflow limitations and increased respiratory effort affecting phonation,^7^,^16^,^17^ clinical studies have reported laryngeal involvement in adults with severe asthma.^42^,^43^ These include laryngeal hypersensitivity, impaired laryngeal control, and functional laryngeal abnormalities affecting phonation or respiration, in line with the concept of unified airways.^44^ Notably, Vertigan et al^43^ found that both patient-reported symptoms and laryngeal dysfunction improved following targeted voice therapy, underscoring the clinical relevance of identifying and addressing voice problems in individuals with asthma. These findings suggest plausible mechanisms through which asthma-related physiological and behavioral factors may be associated with voice problems independently of pharmacological exposure, consistent with the present results.

Chronic cough also had an independent association with voice problems, which is consistent with earlier findings.^19^,^45^ Beyond the mechanical effects of repeated coughing and throat clearing, chronic cough is increasingly understood as a manifestation of laryngeal hypersensitivity, which can itself affect phonatory function.^46^,^47^ These mechanisms suggest that chronic cough may contribute to voice problems alongside ICS exposure and asthma-related factors.

### Clinical Relevance

In the present study, there was substantial overlap between ICS use, asthma symptoms, and chronic cough, and voice problems were most frequent among individuals experiencing more than one of these factors. This pattern suggests that multiple mechanisms such as cough-related laryngeal irritation, ICS-related mucosal effect, and asthma-related airway inflammation act concurrently rather than in isolation. Clinically, this overlap identifies a subgroup at particularly high risk of voice problems and highlights a therapeutic dilemma: in some individuals, improved symptom control may reduce coughing and vocal strain, whereas in others higher ICS doses may exacerbate voice problems through local steroid effects. Accordingly, patients presenting with this combination of factors warrant clinical attention, especially in middle-aged and older adults, for whom susceptibility to voice problems may be greater.^48^ In selected cases, interventions such as voice therapy and cough suppression techniques have the potential to reduce symptom burden.^43^,^49^,^50^

### Strengths and Weaknesses

This study has several strengths. It is one of few population-based studies investigating ICS-related voice problems, allowing prevalence and estimates that are generalizable beyond clinical samples. The multicenter design and use of linked questionnaire and prescription register data strengthen the robustness and generalizability of the findings. The analyses also adjusted for multiple respiratory conditions and lifestyle factors known to influence voice outcomes, increasing confidence that the observed associations with ICS use are not merely markers of other underlying morbidities.

Some limitations should be acknowledged. First, ICS exposure was assessed using dispensed prescriptions during 2021, and the lowest exposure category (1–150 FDDD/year) likely included individuals with minimal or intermittent ICS use. This may have led to exposure misclassification, particularly among participants with very low cumulative dose. In addition, questionnaire data were collected between 2021 and 2022 meaning that dispensed ICS in 2021 may not correspond to treatment status at the time voice problems were reported. Both sources of misclassification are likely non-differential and would tend to attenuate the observed associations.

Another limitation concerns the classification of asthma and asthma symptoms. In line with many population-based studies, asthma was defined as self-reported physician-diagnosed asthma. Although this approach generally shows fair-to-good agreements with clinical records, some misclassification is expected.^51^ In addition, the asthma symptoms items used may not exclusively reflect asthma-related disease activity. Nevertheless, symptom-based definitions of asthma-related morbidity are widely used in population-based research to capture disease activity beyond physician-diagnosed asthma.

Because few participants reported severe voice problems, response categories were merged into a single outcome. While this limits differentiation between levels of symptom severity, even mild voice complaints can have functional consequences, making the binary classification appropriate for analysis.^52^

Loss to follow-up from previous RHINE waves may have introduced nonresponse bias. However, previous evaluations of the RHINE cohort found that even if differences between responders and non-responders could have minor implications for prevalence estimates, they did not affect exposure outcome associations.^20^

## Conclusions

In this population-based study, both ICS use and asthma-related symptoms were independently associated with self-reported voice problems in a dose-dependent manner. Voice problems were particularly common among individuals in whom ICS use, asthma symptoms, and chronic cough co-occurred, underscoring a subgroup with substantial vocal burden. These findings highlight the importance of recognizing and monitoring voice problems in patients with symptomatic airway disease who use ICS and suggest that vocal outcomes should be considered when evaluating and optimizing ICS treatment strategies.

## Data Availability

The datasets analyzed during the current study are not publicly available due to regulatory demands but are available from the corresponding author on reasonable request.

## Abbrevations

AOR, Adjusted odds ratio; BMI, Body mass index; CI, Confidence interval; COPD, Chronic obstructive pulmonary disease; CRS, Chronic rhinosinusitis; DAG, Directed acyclic graph; DPI, Dry-powder inhalers; EPOS, European Position Paper on Rhinosinusitis and Nasal Polyps; FDDD, Filled defined daily doses; ICS, Inhaled corticosteroids; nGER, Nocturnal gastroesophageal reflux; pMDI, pressurized metered-dose inhaler; OR, Odds ratio; RHINE, Respiratory Health in Northern Europe; SD, Standard deviation.

## References

[1] Papi A, Brightling C, Pedersen SE, Reddel HK. Asthma. *Lancet*. 2018;391(10122):783–13. doi:10.1016/s0140-6736(17)33311-1 29273246

[2] Porsbjerg C, Melén E, Lehtimäki L, Shaw D. Asthma. *Lancet*. 2023;401(10379):858–873. doi:10.1016/s0140-6736(22)02125-0 36682372

[3] Oh BC, Lee JE, Nam JH, Hong JY, Kwon SH, Lee EK. Health-related quality of life in adult patients with asthma according to asthma control and severity: a systematic review and meta-analysis. *Front Pharmacol*. 2022;13:908837. doi:10.3389/fphar.2022.908837 PMC9720394 36479200

[4] Global Initiative for Astma (GINA). Global strategy for asthma management and prevention. Available from: https://ginasthma.org/wp-content/uploads/2024/05/GINA-2024-Strategy-Report-24_05_22_WMS.pdf. Accessed April 11, 2026.

[5] Quint JK, Ariel A, Barnes PJ. Rational use of inhaled corticosteroids for the treatment of COPD. *NPJ Prim Care Respir Med*. 2023;33(1):27. doi:10.1038/s41533-023-00347-6 PMC10366209 37488104

[6] Shang W, Wang G, Wang Y, Han D. The safety of long-term use of inhaled corticosteroids in patients with asthma: a systematic review and meta-analysis. *Clin Immunol*. 2022;236:108960. doi:10.1016/j.clim.2022.108960 35218965

[7] Spantideas N, Drosou E, Bougea A, Assimakopoulos D. Inhaled corticosteroids and voice problems. What is new? *J Voice*. 2017;31(3):384.e1–384.e7. doi:10.1016/j.jvoice.2016.09.002 27742496

[8] Ihre E, Zetterström O, Ihre E, Hammarberg B. Voice problems as side effects of inhaled corticosteroids in asthma patients—a prevalence study. *J Voice*. 2004;18(3):403–414. doi:10.1016/j.jvoice.2003.05.003 15331115

[9] Rachelefsky GS, Liao Y, Faruqi R. Impact of inhaled corticosteroid-induced oropharyngeal adverse events: results from a meta-analysis. *Ann Allergy Asthma Immunol*. 2007;98(3):225–238. doi:10.1016/s1081-1206(10)60711-9 17378253

[10] Chmielewska M, Akst LM. Dysphonia associated with the use of inhaled corticosteroids. *Curr Opin Otolaryngol Head Neck Surg*. 2015;23(3):255–259. doi:10.1097/moo.0000000000000153 25887975

[11] Lechien JR. Influence of inhaled corticosteroids on voice quality: a systematic review. *J Voice Apr*. 2025. doi:10.1016/j.jvoice.2025.04.008 40287307

[12] Ivanova JI, Birnbaum HG, Hsieh M, et al. Adherence to inhaled corticosteroid use and local adverse events in persistent asthma. *Am J Manag Care*. 2008;14(12):801–809. 19067497

[13] Persaud PN, Tran AP, Messner D, et al. Perception of burden of oral and inhaled corticosteroid adverse effects on asthma-specific quality of life. *Ann Allergy Asthma Immunol*. 2023;131(6):745–751.e11. doi:10.1016/j.anai.2023.08.595 PMC10843134 37643678

[14] Fitzgerald JM, Chan CK, Holroyde MC, Boulet LP. The CASE survey: patient and physician perceptions regarding asthma medication use and associated oropharyngeal symptoms. *Can Respir J*. 2008;15(1):27–32. doi:10.1155/2008/593723 PMC2677852 18292850

[15] Park B, Choi HG. Association between asthma and dysphonia: a population-based study. *J Asthma*. 2016;53(7):679–683. doi:10.3109/02770903.2016.1140181 27186798

[16] Asnaashari AM, Rezaei S, Babaeian M, et al. The effect of asthma on phonation: a controlled study of 34 patients. *Ear Nose Throat J*. 2012;91(4):168–171. doi:10.1177/014556131209100409 22522355

[17] Dogan M, Eryuksel E, Kocak I, Celikel T, Sehitoglu MA. Subjective and objective evaluation of voice quality in patients with asthma. *J Voice*. 2007;21(2):224–230. doi:10.1016/j.jvoice.2005.11.003 16504474

[18] Çolak Y, Afzal S, Lange P, Laursen LC, Nordestgaard BG, Dahl M. Role and impact of chronic cough in individuals with asthma from the general population. *J Allergy Clin Immunol*. 2019;7(6):1783–1792.e8. doi:10.1016/j.jaip.2019.02.021 30836227

[19] Ögefeldt S, Wistbacka G, Schalling E, et al. Voice problems in chronic cough: prevalence and implications for health and sick leave in a Northern European population. *BMC Pulm Med*. 2025;25(1):402. doi:10.1186/s12890-025-03877-6 PMC12369044 40841619

[20] Johannessen A, Verlato G, Benediktsdottir B, et al. Longterm follow-up in European respiratory health studies - patterns and implications. *BMC Pulm Med*. 2014;14:63. doi:10.1186/1471-2466-14-63 PMC4021078 24739530

[21] Torén K, Gislason T, Omenaas E, et al. A prospective study of asthma incidence and its predictors: the RHINE study. *Eur Respir J*. 2004;24(6):942–946. doi:10.1183/09031936.04.00044804 15572536

[22] Lyberg-åhlander V, Rydell R, Fredlund P, Magnusson C, Wilén S. Prevalence of voice disorders in the general population, based on the Stockholm public health cohort. *J Voice*. 2019;33(6):900–905. doi:10.1016/j.jvoice.2018.07.007 30126692

[23] RHINE. Welcome to RHINE – a research network studying Respiratory Health in Northern Europe. Available from: https://rhine.w.uib.no/. Accessed December 3, 2024.

[24] WHO Collaborating Centre for Drug Statistics Methodology. Guidelines for ATC classification and DDD assignment. Available from: https://atcddd.fhi.no/atc_ddd_index/. Accessed April 12, 2026.

[25] Bjerg A, Ekerljung L, Middelveld R, et al. Increased prevalence of symptoms of rhinitis but not of asthma between 1990 and 2008 in Swedish adults: comparisons of the ECRHS and GA^2^LEN surveys. *PLoS One*. 2011;6(2):e16082. doi:10.1371/journal.pone.0016082 PMC3040758 21379386

[26] ECRHS. Variations in the prevalence of respiratory symptoms, self-reported asthma attacks, and use of asthma medication in the European Community Respiratory Health Survey (ECRHS). *Eur Respir J*. 1996;9(4):687–695. doi:10.1183/09031936.96.09040687. 8726932

[27] de Marco R, Cerveri I, Bugiani M, Ferrari M, Verlato G. An undetected burden of asthma in Italy: the relationship between clinical and epidemiological diagnosis of asthma. *Eur Respir J*. 1998;11(3):599–605. 9596109

[28] Johansson H, Johannessen A, Holm M, et al. Prevalence, progression and impact of chronic cough on employment in Northern Europe. *Eur Respir J*. 2021;57(6):2003344. doi:10.1183/13993003.03344-2020 33303532

[29] Gislason T, Janson C, Vermeire P, et al. Respiratory symptoms and nocturnal gastroesophageal reflux: a population-based study of young adults in three European countries. *Chest*. 2002;121(1):158–163. doi:10.1378/chest.121.1.158 11796445

[30] Fokkens WJ, Lund VJ, Hopkins C, et al. European position paper on rhinosinusitis and nasal polyps 2020. *Rhinology*.;58(Suppl S29):1–464. doi:10.4193/Rhin20.600. 32077450

[31] Wang J, Janson C, Malinovschi A, et al. Asthma, allergic rhinitis and atopic dermatitis in association with home environment - The RHINE study. *Sci Total Environ*. 2022;853:158609. doi:10.1016/j.scitotenv.2022.158609 36089044

[32] Naunheim MR, Huston MN, Bhattacharyya N. Voice disorders associated with the use of inhaled corticosteroids. *Otolaryngol Head Neck Surg*. 2023;168(5):1034–1037. doi:10.1002/ohn.198 36939522

[33] Ishizuka T, Hisada T, Aoki H, et al. Gender and age risks for hoarseness and dysphonia with use of a dry powder fluticasone propionate inhaler in asthma. *Allergy Asthma Proc*. 2007;28(5):550–556. doi:10.2500/aap2007.28.3049 18034974

[34] Hassen HE, Abo Hasseba AM. Voice evaluation in asthma patients using inhaled corticosteroids. *Kulak Burun Bogaz Ihtis Derg*. 2016;26(2):101–108. doi:10.5606/kbbihtisas.2016.79740 26890712

[35] Buhl R. Local oropharyngeal side effects of inhaled corticosteroids in patients with asthma. *Allergy*. 2006;61(5):518–526. doi:10.1111/j.1398-9995.2006.01090.x 16629778

[36] Jacobson BH, Johnson A, Grywalski C, et al. The voice handicap index (VHI) development and validation. *Am J Speech Lang Pathol*. 1997;6(3):66–70.

[37] Adams NP, Bestall JC, Jones PW, Lasserson TJ, Griffiths B, Cates C. Inhaled fluticasone at different doses for chronic asthma in adults and children. *Cochrane Database Syst Rev*. 2005;(3):Cd003534. doi:10.1002/14651858.CD003534.pub2 16034902

[38] Dubus JC, Marguet C, Deschildre A, et al. Local side-effects of inhaled corticosteroids in asthmatic children: influence of drug, dose, age, and device. *Allergy*. 2001;56(10):944–948. doi:10.1034/j.1398-9995.2001.00100.x 11576072

[39] Ozbilen Acar G, Uzun Adatepe N, Kaytaz A, et al. Evaluation of laryngeal findings in users of inhaled steroids. *Eur Arch Otorhinolaryngol*. 2010;267(6):917–923. doi:10.1007/s00405-009-1141-2 19908054

[40] Williams AJ, Baghat MS, Stableforth DE, Cayton RM, Shenoi PM, Skinner C. Dysphonia caused by inhaled steroids: recognition of a characteristic laryngeal abnormality. *Thorax*. 1983;38(11):813–821. doi:10.1136/thx.38.11.813 PMC459669 6648863

[41] Lavy JA, Wood G, Rubin JS, Harries M. Dysphonia associated with inhaled steroids. *J Voice*. 2000;14(4):581–588. doi:10.1016/s0892-1997(00)80014-4 11130115

[42] Migueres N, Delmas C, Petit Thomas J, et al. Laryngeal dysfunction is prominent in asthmatic women treated by inhaled corticosteroids. *Clin Transl Allergy*. 2022;12(12):e12211. doi:10.1002/clt2.12211 PMC9734276 36573313

[43] Vertigan AE, Kapela SL, Gibson PG. Laryngeal dysfunction in severe asthma: a cross-sectional observational study. *J Allergy Clin Immunol*. 2021;9(2):897–905. doi:10.1016/j.jaip.2020.09.034 33011304

[44] Krouse JH, Altman KW. Rhinogenic laryngitis, cough, and the unified airway. *Otolaryngol Clin North Am*. 2010;43(1):111–121. doi:10.1016/j.otc.2009.11.005 20172261

[45] Vertigan AE, Theodoros DG, Winkworth AL, Gibson PG. Perceptual voice characteristics in chronic cough and paradoxical vocal fold movement. *Folia Phoniatr Logop*. 2007;59(5):256–267. doi:10.1159/000104464 17726329

[46] Chung KF. Chronic ‘cough hypersensitivity syndrome’: a more precise label for chronic cough. *Pulm Pharmacol Ther*. 2011;24(3):267–271. doi:10.1016/j.pupt.2011.01.012 21292019

[47] Cobeta I, Pacheco A, Mora E. The role of the larynx in chronic cough. *Acta Otorrinolaringol Esp*. 2013;64(5):363–368. doi:10.1016/j.otoeng.2013.10.007 23305863

[48] Wang LH, Doan TN, Chang FC, To TL, Ho WC, Chou LW. Prevalence of voice disorders in older adults: a systematic review and meta-analysis. *Am J Speech Lang Pathol*. 2023;32(4):1758–1769. doi:10.1044/2023_ajslp-22-00393 37285381

[49] Chamberlain Mitchell SAF, Garrod R, Clark L, et al. Physiotherapy, and speech and language therapy intervention for patients with refractory chronic cough: a multicentre randomised control trial. *Thorax*. 2017;72(2):129–136. doi:10.1136/thoraxjnl-2016-208843 27682331

[50] Vertigan AE, Theodoros DG, Gibson PG, Winkworth AL. Efficacy of speech pathology management for chronic cough: a randomised placebo controlled trial of treatment efficacy. *Thorax*. 2006;61(12):1065–1069. doi:10.1136/thx.2006.064337 PMC2117063 16844725

[51] Rousseau MC, Conus F, El-Zein M, Benedetti A, Parent ME. Ascertaining asthma status in epidemiologic studies: a comparison between administrative health data and self-report. *BMC Med Res Methodol*. 2023;23(1):201. doi:10.1186/s12874-023-02011-6 PMC10486089 37679673

[52] DeVore EK, Shrime MG, Wittenberg E, Franco RA, Song PC, Naunheim MR. The health utility of mild and severe dysphonia. *Laryngoscope*. 2020;130(5):1256–1262. doi:10.1002/lary.28216 31381164

